# Association between Pre-stroke Frailty and Clinical Outcomes: A Systematic Review and Meta-analysis

**DOI:** 10.1298/ptr.25-E10357

**Published:** 2025-09-11

**Authors:** Issei MURAI, Kensuke MATSUDA, Takashi ARIIE

**Affiliations:** 1 Department of Rehabilitation, Takagi Hospital, Japan; 2 Department of Physical Therapy, School of Health Sciences at Fukuoka, International University of Health and Welfare, Japan

**Keywords:** Frailty, Stroke, Mortality, Length of stay, Functional outcomes

## Abstract

**Objective:**

The relationship between pre-stroke frailty and clinical outcomes remains unclear. This systematic review aimed to examine the association of pre-stroke frailty with mortality, length of stay (LOS), and functional outcomes in people with stroke.

**Methods:**

We used several databases, including PubMed, EMBASE, and CENTRAL. We searched for studies investigating the association between pre-stroke frailty and clinical outcomes. The Quality in Prognosis Studies tool was used to assess the risk of bias in the included studies. Meta-analyses were performed using the random effects model. The certainty of evidence was assessed with the Grading of Recommendations, Assessment, Development, and Evaluation.

**Results:**

Fourteen studies (participants: 11583) were included in this review. Pre-stroke frailty is associated with higher mortality (odds ratio: 1.11; 95% confidence intervals [CI]: 1.0–1.23), longer LOS (mean difference: 0.75; 95% CI: −0.29 to 1.78), and poorer functional outcomes (standardized mean difference: 0.79; 95% CI: 0.48–1.1). The certainty of evidence is low due to risk of bias, inconsistency, and imprecision.

**Conclusions:**

These results suggest that frailty before stroke onset may be associated with higher mortality, increased LOS, and poorer functional outcomes.

## Introduction

Frailty, a health condition prevalent among older individuals, has recently received increasing attention from the perspective of extending healthy life expectancy. The ADVANTAGE Joint Action of the World Health Organization and the European Union defines frailty as a progressive decline in physiological systems associated with aging^1)^. Frailty leads to reduced innate ability reserves, heightened vulnerability to stressors, and an increased risk of various health complications^2)^. These vulnerabilities can cause health problems, including disability, loss of independence, and death, suggesting that frailty should be regarded as a multidimensional concept^1)^.

Frailty is associated with various disease outcomes. For example, frailty in people with cancer has adverse effects on postoperative complications, short- and long-term postoperative mortality, and length of stay (LOS)^3)^. Furthermore, an association between frailty and increased mortality has been reported in older people with heart failure^4)^. Therefore, identifying frailty in older people is essential for a comprehensive approach to addressing these disabling conditions^5)^. The prevalence rate of frailty before stroke onset was high (approximately 25%) in a previous systematic review^6)^. This rate is higher than that observed in community-dwelling older people, which is 7%–10%^7)^. However, the association between pre-stroke frailty and clinical outcomes remains unclear.

Predicting outcomes in people with acute stroke remains challenging^8)^, and a better understanding of the association between pre-stroke frailty and clinical outcomes, such as mortality, LOS, and functional outcomes, may provide prognostic insights. Mortality and functional outcomes are considered core outcome sets for people with stroke^9)^, and LOS is an important indicator of healthcare costs^10)^. The previous systematic review examined the association between pre-stroke frailty and mortality; however, the results were based on a qualitative synthesis and did not assess the certainty of the evidence^6)^. In addition, several primary studies have been reported since the publication of the previous systematic review^11,12)^. Including these studies in a quantitative synthesis would provide a better estimate of the association between frailty before stroke onset and clinical outcomes. The aim of this systematic review was to examine the association of pre-stroke frailty with mortality, LOS, and functional outcomes.

## Methods

This systematic review and meta-analysis were reported in accordance with the Meta-analysis of Observational Studies in Epidemiology (MOOSE guidelines)^13)^ (Supplementary Table 1). The protocol was pre-registered in PROSPERO (CRD:42024552097). We followed the Cochrane Handbook for the method development^14)^.

### Eligibility criteria

We included observational studies (cohort studies, case–control studies, or secondary analyses of clinical trials) without any restriction on publication status or language. Case reports or case series were excluded. Studies were eligible for inclusion if they recruited adult participants aged 18 years or older with a diagnosis of stroke, regardless of stroke subtype, except for transient ischemic attack and subarachnoid hemorrhage. Exposures were frailty before stroke onset, including those judged by binary assessments, those using categorical ratings (e.g., frail, pre-frailty, or non-frailty), and those using continuous variables such as the Frailty Index^15)^. We also included other assessments for frailty. Comparisons were non-frailty as defined by the authors of the original studies. The following 3 outcomes were assessed: mortality, LOS, and functional outcomes. If multiple outcome measurements were reported for functional outcomes, the modified Rankin Scale (mRS), Barthel Index (BI), and other outcomes were prioritized in this order. Data with the longest follow-up period were used if multiple time points of the outcome assessments were available.

### Search strategy

We searched PubMed, EMBASE via Dialog, the Cochrane CENTRAL, CINAHL via EBSCO, the World Health Organization International Clinical Trials Registry Platform (ICTRP), and ClinicalTrials.gov on June 14, 2024. Search terms such as “stroke” and “frailty” were used (Supplementary Tables 2–7). We also reviewed the reference lists of the included studies and the studies citing the included studies.

### Selection process and data extraction

Two independent reviewers (IM and KM) screened the titles and abstracts of the identified articles. Subsequently, a full-text screening of the remaining articles was performed to check the eligibility for this review. Two reviewers (IM and KM) independently extracted data from the included studies using a pre-developed data extraction form. The data included study characteristics (e.g., authors, year of publication, country, study design, and method of determining frailty) and results (e.g., sample size and outcome). Conflicts regarding the results of screening and data extraction between the reviewers were resolved through discussion. If a conflict persisted, a third reviewer (TA) joined and resolved it.

### Risk of bias assessment

Two reviewers (IM and KM) independently assessed the risk of bias in the included studies using the Quality in Prognosis Studies (QUIPS) tool^16)^. The QUIPS tool has 6 domains: study participation, study attrition, prognostic factor measurement, outcome measurement, study confounding, and statistical analysis/reporting. Each domain was scored as high, moderate, or low risk of bias. We predefined the following confounders: age, sex, blood pressure, stroke severity, race, and body mass index based on previous studies^17,18)^.

### Statistical analysis

We used the odds ratio (OR) with a 95% confidence interval (95% CI) for the binary outcome (mortality), and the mean difference (MD) and standardized mean difference (SMD) with 95% CI for continuous outcomes (LOS and functional outcomes, respectively). A meta-analysis was performed using a random-effects model in RevMan 5.4 (Cochrane Collaboration, London, UK). The adjusted OR (aOR) was prioritized in the main analysis. A crude OR was used when an aOR was unavailable.

Heterogeneity was assessed by visual inspection of the forest plot and using I^2^ statistics (I^2^ values of 0%–40%: might not be important; 30%–60%: may represent moderate heterogeneity; 50%–90%: may represent substantial heterogeneity; and 75%–100%: considerable heterogeneity)^11)^. We also calculated the Cochran χ^2^ test (Q test), with a P value of 0.10 being statistically significant. If heterogeneity existed (I^2^ >50%), the possible reasons for heterogeneity were evaluated using the subgroup analyses based on stroke type (cerebral hemorrhage versus cerebral infarction) and age (≥65 years old versus <65 years).

### Missing data

The authors of the included studies were contacted to address missing data or any uncertainties. The analysis used the available data if the authors could not be contacted.

### Sensitivity analysis

The following sensitivity analyses were performed:

1. Excluding studies that reported crude ORs only.
2. Excluding studies with a high risk of bias in the overall quality.

### Assessment of evidence certainty

The certainty of evidence for each outcome was assessed using the Grading of Recommendations, Assessment, Development, and Evaluations (GRADE) approach^19)^, and the results were described according to an informative statement^20)^. The GRADE items included study limitations, data inconsistency, indirectness of evidence, data imprecision, and publication bias. ClinicalTrials.gov and ICTRP were searched to identify unpublished studies. If more than 10 studies were found for a single outcome, a visual inspection of the funnel plot and Egger’s test was performed (P <0.05).

## Results

**Fig. 1.**
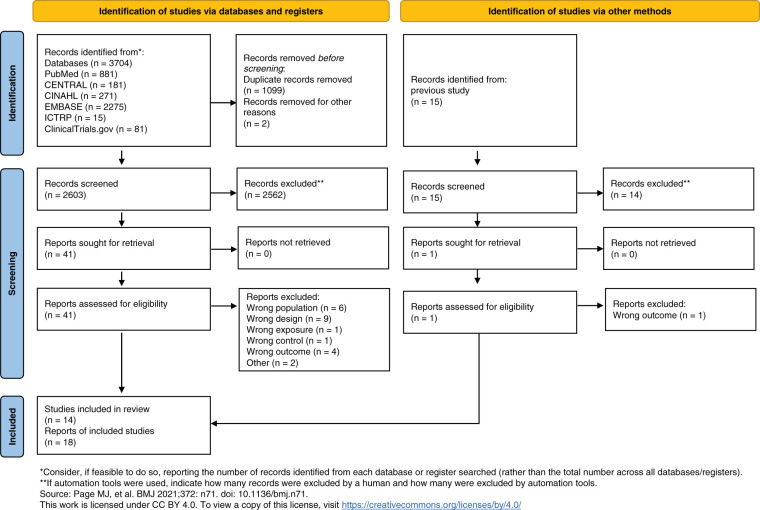
Preferred Reporting Items for Systematic Reviews and Meta-Analyses (PRISMA) Flowchart

The study selection process is illustrated in Fig. 1. Studies excluded during the full-text screening are listed with reasons in Supplementary Table 8. The database search identified 3704 studies, of which 14 studies^7,21–33)^ involving 11583 participants were included in this review.

### Study characteristics (Table 1)

**Table 1.** Characteristics of the included studies

Author (year)	Research design	Study participants (recruitment criteria, stroke subtype, age, sample size)	Frailty evaluation method	Outcome(s)
Mennema et al. (2023)^21)^	Prospective cohort	Patients diagnosed with stroke within the past 6 months, ischemic stroke/cerebral hemorrhage, median age: 70 (IQR 6), n = 322	GFI	• LOS • BI at discharge
			Frail ≧4, non-frail <4	
Joyce et al. (2022)^22)^	Prospective cohort	Patients undergoing MT, ischemic stroke, mean 72 (SD 13), n = 175	Frailty index	• Mortality at 90 days after MT • mRS at 90 days after MT
			Frailty ≧0.24	
Yang et al. (2022)^23)^	Prospective cohort	Patients with acute stroke, ischemic stroke, non-frailty: median 74 (IQR 2)/frailty: median 78 (IQR 14), n = 215	FRAIL scale	• Mortality at 1 year after onset • mRS at 1 year after onset
			Frailty = 3–5, robust = 1	
Pilotto et al. (2022)^24)^	Prospective cohort	Patients treated for reperfusion, ischemic stroke, mean 77.2 (SD 6.7), n = 102	MPI	• Mortality at 1 year after onset • mRS at 1 year after onset
			Frailty ≧0.34, robust <0.33	
Schnieder et al. (2021)^25)^	Case–control study	LVOS and endovascularly treated patients, ischemic stroke, median 80.1 (IQR 9.58), n = 433	HFRS	• Mortality at 90 days after onset • LOS • mRS at 90 days after onset
			Low <5, intermediate = 5–15	
			High >15	
Evans et al. (2020)^26)^	Prospective cohort	Ischemic stroke, non-frailty: median 83 (IQR 9)/frailty: median 87 (IQR 9), n = 433	CFS	• Mortality at 28 days after onset
			Frailty = 5–8, non-frailty = 1–4	
O’Caoimh et al. (2024)^27)^	Prospective cohort	Patients with cardiogenic ischemic stroke due to AF, median 80 (IQR 14), n = 113	CFS	• Mortality at 30 days after onset • LOS • mRS at 30 days after onset
			Frailty = 5–8, non-frailty = 1–4	
Tiainen et al. (2022)^28)^	Case–control study	Patients over 80 years of age undergoing EVT, ischemic stroke, median 83 (IQR 5), n = 159	CFS	• Mortality at 1 year after EVT
			Frailty = 5–9, non-frailty = 1–4	
Iwasawa et al. (2023)^7)^	Prospective cohort	Acute stroke patients, ischemic stroke/cerebral hemorrhage, mean 79.2 (SD 7.4), n = 210	CFS	• mRS at discharge
			Frailty = 5–8, non-frailty = 1–4	
Tan et al. (2022)^29)^	Case–control study	Acute ischemic stroke patients over 70 years of age undergoing EVT, mean 78.1 (SD 5.7), n = 198	CFS	• Mortality at 90 days after onset • mRS at 90 days after onset
			Frailty >3, non-frailty = 1–3	
Seamon and Simpson (2019)^30)^	Case–control study	Acute stroke, ischemic stroke, mean 79.4 (SD 8.4), n = 7258	Faurot Frailty Index	• LOS
			Non-frailty <0.1, frailty >5.0	
Noguchi, et al. (2021)^31)^	Prospective cohort	Acute stroke patients 65 years and older, ischemic stroke/cerebral hemorrhage, median 76 (IQR 11), n = 232	SFI	• LOS • mRS at discharge
			Frailty = 0, frailty ≧3	
Miranda et al. (2022)^32)^	Prospective cohort	Acute ischemic stroke patients 40 years and older, mean 69 (SD 13), n = 174	PRISMA-7	• LOS
			Frailty = 3	
Schnieder et al. (2022)^33)^	Prospective cohort	LVOS and endovascularly treated patients, ischemic stroke/cerebral hemorrhage, n = 1559	HFRS	• Mortality at 90 days after onset
			Low <5, intermediate = 5–15	
			High >15	

IQR: interquartile range, GFI: groningen frailty indicator, LOS: length of stay, BI: barthel index, MT: mechanical thrombectomy, SD: standard deviation, mRS: modified rankin scale, MPI: multiple prognostic index, LVOS: large vessel occlusion scale, HFRS: hospital frailty risk score, CFS: clinical frailty scale, AF: atrial fibrillation, EVT: endovascular therapy, SFI: simplified frailty index, nr: not reported

The median of the mean ages among the included studies was 78.1 years. The study designs were prospective cohort studies (10 studies), and case–control studies (4 studies). Ten studies included only participants with cerebral infarction, and 4 included participants with both cerebral infarction and hemorrhage. Other details of the study characteristics are shown in Supplementary Table 9.

### Risk of bias assessment of included studies (Supplementary Table 10)

The risk of bias assessment using the QUIPS tool showed that 5 studies were rated as high, 7 as low, and 2 as moderate in overall quality. More than half of the included studies (64%) had a moderate-to-high risk of bias in the study attrition domain.

### Frailty assessment

The tools used to assess frailty varied across the included studies: 5 studies used the Clinical Frailty Scale, 2 used the Hospital Frailty Risk Score, and others used the Frailty Index, FRAIL scale, multiple prognostic impairment, Groningen Frailty Indicator, Faurot Frailty Index, Simplified Frailty Index, and PRISMA-7 in 1 study each.

### Pre-stroke frailty and clinical outcomes

The effect estimates and certainty of evidence regarding the association between pre-stroke frailty and each clinical outcome are summarized in Table 2.

**Table 2.** Summary of the association between pre-stroke frailty and clinical outcomes

Outcomes	Anticipated absolute effects (95% CI)^a^				
	Assumed risk with control^b^	Corresponding risk with pre-stroke frailty	Relative effect (95% CI)	No. of participants (studies)	Certainty of evidence (GRADE)
Mortality	Study population 112 per 1000	124.3 Per 1000 (112–137.8)	OR 1.11 (1–1.23)	2968 (9 studies)	⊕⊕⊖⊖ Low^c,d^
LOS	Study population	MD 0.75 (−0.29 to 1.78)		6027 (8 studies)	⊕⊕⊖⊖ Low^c,e^
Functional outcome	Study population	SMD 0.79 (0.48–1.1)		1594 (9 studies)	⊕⊕⊖⊖ Low^c,e^

**GRADE Working Group grades of evidence**: **High certainty**: we are very confident that the true effect lies close to that of the estimate of the effect. **Moderate certainty**: we are moderately confident in the effect estimate: the true effect is likely to be close to the estimate of the effect, but there is a possibility that it is substantially different. **Low certainty**: our confidence in the effect estimate is limited: the true effect may be substantially different from the estimate of the effect. **Very low certainty**: we have very little confidence in the effect estimate: the true effect is likely to be substantially different from the estimate of the effect.

^a^The corresponding risk (95% CI) was based on the assumed risk of the nonexposed group and the relative effect of exposure (95% CI).

^b^Median event rate of the included studies.

^c^Downgraded by one level for study limitations: assessed with the QUIPS tool (moderate or high risk of bias in the domain).

^d^Downgraded by one level for inconsistency: the included studies showed differing directions of point estimates.

^e^Downgraded by one level for imprecision: judged by confidence interval (confidence interval crossed zero).

LOS, length of stay; CI, confidence interval; OR: odds ratio; MD, mean difference; SMD, standardized mean difference; GRADE, Grading of Recommendations, Assessment, Development, and Evaluations; QUIPS, Quality in Prognosis Studies

**Fig. 2.**
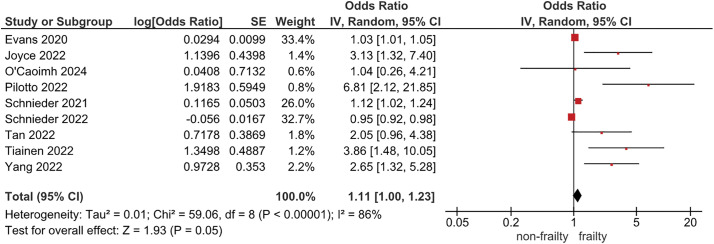
Forest plot of mortality SE, standard error; CI, confidence interval

For the association between pre-stroke frailty and mortality, 9 studies were included in the meta-analysis (Fig. 2). The median follow-up period was 90 days (range, 28–365 days). The results suggest that pre-stroke frailty may be associated with higher mortality (OR: 1.11; 95% CI: 1.0–1.23; I^2^ = 86%; n = 2968; certainty of evidence: low).

**Fig. 3.**
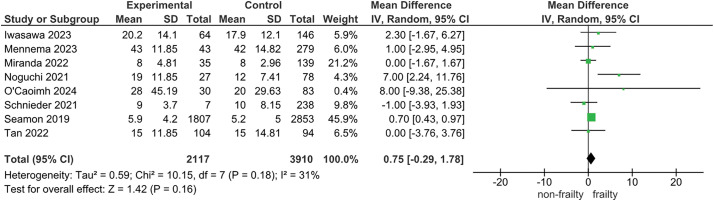
Forest plot of length of stay SD, standard deviation; CI, confidence interval

For the association between pre-stroke frailty and LOS, 8 studies were included in the meta-analysis (Fig. 3). The median LOS was 17 days (range, 5–43 days) in the frail group and 13.5 days (range, 5–42 days) in the non-frailty group. The results suggest that pre-stroke frailty may be associated with a longer hospital stay (MD: 0.75; 95% CI: −0.29 to 1.78; I^2^ = 31%; n = 6027; certainty of evidence: low).

**Fig. 4.**
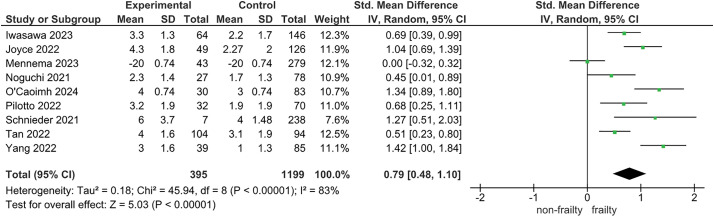
Forest plot of functional outcome CI, confidence interval; SD, standard deviation

For the association between pre-stroke frailty and functional outcome, 9 studies were included in the meta-analysis (Fig. 4). The median follow-up period was 90 days (range, 30–365 days). The outcome measures were mRS in 8 studies and BI in 1. The results suggest that pre-stroke frailty may be associated with poor functional outcome (SMD: 0.79; 95% CI: 0.48–1.1; I^2^ = 83%; n = 1594; certainty of evidence: low).

### Additional analysis

Substantial heterogeneity was observed in the analyses of mortality (I^2^ = 86%) and functional outcomes (I^2^ = 83%). However, subgroup analyses could not be conducted because the mean age of the participants was 65 years or older in all studies, and none of the studies included only participants with cerebral hemorrhage.

At least 1 predefined sensitivity analysis was performed for each association, excluding studies that reported crude ORs and those with a high risk of bias. All analyses revealed consistent associations between pre-stroke frailty and the respective outcomes (Supplementary Figs. 1–4).

### Publication bias

Publication bias was not assessed because fewer than 10 studies were included for each outcome.

## Discussion

This systematic review aimed to investigate the association between pre-stroke frailty and mortality, LOS, and functional outcomes in participants with stroke. Fourteen studies involving 11583 participants were included in the analysis. The findings suggest that pre-stroke frailty may be associated with higher mortality, increased LOS, and poor functional outcomes. However, the certainty of evidence was low across the outcomes. The reason for this downgrade is mainly the limitations of the included studies, in particular, high study attrition and inadequately controlled confounding factors. Further research should address these points by implementing robust follow-up systems or collecting comprehensive variables for confounders, for instance, to enhance the certainty of the evidence.

Frailty before stroke onset may be associated with higher mortality. The clinical manifestations of frailty are observed during continuous dysregulation, with increased mortality and poor functional outcomes^34)^. Stress events in people with frailty rapidly reduce function and capacity^35)^ and increase the risk of acute illness, mortality, and severity^36)^. It is reported that mortality from ischemic stroke has declined^37)^. However, the prevalence of frailty has increased among older people^38)^. Further research may be needed to investigate effective approaches to decrease the mortality in this population.

In our review, longer hospital stays and lower functional outcomes may also be associated with pre-stroke frailty. For increased LOS, the results are consistent with a previous review in a surgical population^39)^. Another review reported that patients with frailty are discharged to healthcare facilities more often than those without^40)^. This may reflect the need for continuous medical support in the frailty population. Furthermore, an observational study reported that LOS is a mediator between pre-stroke frailty and poor functional outcomes^31)^. Therefore, pre-stroke frailty, as identified in this study, may be associated with an increased LOS and may mediate poor functional outcomes. People with frailty and stroke have a high incidence of complications, such as urinary tract infections or pneumonia^41)^. Additionally, neurological impairment after stroke onset may exacerbate the clinical symptoms of frailty, such as dysphagia^42)^, which could increase LOS and lead to poor functional outcomes.

This study had several strengths. To the best of our knowledge, this is the first systematic review to assess the certainty of evidence for the association using the GRADE approach. In addition, we followed transparent reporting and rigorous methods in accordance with the MOOSE guidelines and the Cochrane Handbook.

On the other hand, this study also had several limitations. First, substantial heterogeneity was observed between pre-stroke frailty and mortality and functional outcomes. We could not conduct subgroup analysis by prespecified stroke subtype and age due to an insufficient number of studies. Further investigation may be needed to reveal factors that contribute to heterogeneity. Second, although the study analyzed the association between frailty and mortality before stroke onset, the detailed causes of death could not be investigated. The extent to which frailty reflects the loss of cerebrovascular reserve necessary to survive an ischemic attack remains unclear^23)^.

## Conclusions

In conclusion, frailty before stroke onset may be associated with increased mortality, LOS, and poor functional outcomes. Further well-designed studies investigating specific stroke subtypes or younger generations may improve the estimation and reduce the heterogeneity.

## Supplementary Materials

Supplementary Table 1: Checklist of MOOSE Guidelines. Supplementary Table 2: Search strategy (MEDLINE via PubMed). Supplementary Table 3: Search strategy (EMBASE via Dialog). Supplementary Table 4: Search strategy (CENTRAL). Supplementary Table 5: Search strategy (CINAHL via EBSCO). Supplementary Table 6: Search strategy (ICTRP). Supplementary Table 7: Search strategy (Clinicaltrials.gov). Supplementary Table 8: Excluded studies and reasons for exclusion. Supplementary Table 9: Confounders of the included studies. Supplementary Table 10: Risk of bias assessment in studies included in the meta-analyses. Supplementary Figure 1: Sensitivity analysis of the association between pre-stroke frailty and mortality excluding studies that reported crude ORs. Supplementary Figure 2: Sensitivity analysis of the association between pre-stroke frailty and mortality excluding studies reporting a high overall risk of bias. Supplementary Figure 3: Sensitivity analysis of the association between pre-stroke frailty and LOS excluding studies reporting a high overall risk of bias. Supplementary Figure 4: Sensitivity analysis of the association between pre-stroke frailty and functional outcome excluding studies reporting a high overall risk of bias.
