# Supplementary material for: Association between Pre-stroke Frailty and Clinical Outcomes: A Systematic Review and Meta-analysis
Source: Phys Ther Res. 2025 Sep 11;28(3):231–8. doi: 10.1298/ptr.25-E10357 (PMC12778358; doi:10.1298/ptr.25-E10357)
Supplement: Supplementary file 1 — Supplementary Table 1: Checklist of MOOSE Guidelines. Supplementary Table 2: Search strategy (MEDLINE via PubMed). Supplementary Table 3: Search strategy (EMBASE via Dialog). Supplementary Table 4: Search strategy (CENTRAL). Supplementary Table 5: Search strategy (CINAHL via EBSCO). Supplementary Table 6: Search strategy (ICTRP). Supplementary Table 7: Search strategy (Clinicaltrials.gov). Supplementary Table 8: Excluded studies and reasons for exclusion. Supplementary Table 9: Confounders of the included studies. Supplementary Table 10: Risk of bias assessment in studies included in the meta-analyses. Supplementary Figure 1: Sensitivity analysis of the association between pre-stroke frailty and mortality excluding studies that reported crude ORs. Supplementary Figure 2: Sensitivity analysis of the association between pre-stroke frailty and mortality excluding studies reporting a high overall risk of bias. Supplementary Figure 3: Sensitivity analysis of the association between pre-stroke frailty and LOS excluding studies reporting a high overall risk of bias. Supplementary Figure 4: Sensitivity analysis of the association between pre-stroke frailty and functional outcome excluding studies reporting a high overall risk of bias. [file ptr-28-231-s001.pdf]

Supplementary materials

Supplementary Table1: Checklist of MOOSE Guidelines
Supplementary Table2: Search strategy (MEDLINE via PubMed)
Supplementary Table3: Search strategy (EMBASE via Dialog)
Supplementary Table4: Search strategy (CENTRAL)
Supplementary Table5: Search strategy (CINAHL via EBSCO)
Supplementary Table6: Search strategy (ICTRP)
Supplementary Table7: Search strategy (Clinicaltrials.gov)
Supplementary Table8: Excluded studies and reasons for exclusion
Supplementary Table9: Confounders of the included studies
Supplementary Table10: Risk of bias assessment in studies included in the meta-analyses
Supplementary Figure1: Sensitivity analysis of the association between pre-stroke frailty and mortality excluding studies that reported crude ORs
Supplementary Figure2: Sensitivity analysis of the association between pre-stroke frailty and mortality excluding studies reporting a high overall risk of bias
Supplementary Figure3: Sensitivity analysis of the association between pre-stroke frailty and LOS excluding studies reporting a high overall risk of bias
Supplementary Figure4: Sensitivity analysis of the association between pre-stroke frailty and functional outcome excluding studies reporting a high overall risk of bias

Supplementary Table1: Checklist of MOOSE Guidelines

Item No	Recommendation	Reported on Page No
Reporting of background should include		
1	Problem definition	2
2	Hypothesis statement	2
3	Description of study outcome(s)	5-7
4	Type of exposure or intervention used	3
5	Type of study designs used	3
6	Study population	3
Reporting of search strategy should include		
7	Qualifications of searchers (eg, librarians and investigators)	4
8	Search strategy, including time period included in the synthesis and key words	3, Supplementary Table 2-7
9	Effort to include all available studies, including contact with authors	3-5
10	Databases and registries searched	3-4, Supplementary Table 2-7
11	Search software used, name and version, including special features used (eg, explosion)	3-4, Supplementary Table 2-7
12	Use of hand searching (eg, reference lists of obtained articles)	3-4
13	List of citations located and those excluded, including justification	5, Supplementary Table 8
14	Method of addressing articles published in languages other than English	3-4
15	Method of handling abstracts and unpublished studies	3-4
16	Description of any contact with authors	5
Reporting of methods should include		
17	Description of relevance or appropriateness of studies assembled for assessing the hypothesis to be tested	3-4
18	Rationale for the selection and coding of data (eg, sound clinical principles or convenience)	3-4
19	Documentation of how data were classified and coded (eg, multiple raters, blinding and interrater reliability)	3-4
20	Assessment of confounding (eg, comparability of cases and controls in studies where appropriate)	4
21	Assessment of study quality, including blinding of quality assessors, stratification or regression on possible predictors of study results	4-5
22	Assessment of heterogeneity	4-5
23	Description of statistical methods (eg, complete description of fixed or random effects models, justification of whether the chosen models account for predictors of study results, dose-response models, or cumulative meta-analysis) in sufficient detail to be replicated	4-5
24	Provision of appropriate tables and graphics	3-5, Table 1, Figure 1-4, Supplementary Table 1-10
Reporting of results should include		
25	Graphic summarizing individual study estimates and overall estimate	Table 2, Figure 2-4

26	Table giving descriptive information for each study included	Table 1, Supplementary Table 8-9
27	Results of sensitivity testing (eg, subgroup analysis)	7, Supplementary Figure 1-4
28	Indication of statistical uncertainty of findings	5-7, Table 2, Figure 2-4
Reporting of discussion should include		
29	Quantitative assessment of bias (eg, publication bias)	8-9, Supplementary Table 10
30	Justification for exclusion (eg, exclusion of non-English language citations)	Supplementary Table 8
31	Assessment of quality of included studies	8-9, Table 2, Supplementary Table 9-10
Reporting of conclusions should include		
32	Consideration of alternative explanations for observed results	8-9
33	Generalization of the conclusions (ie, appropriate for the data presented and within the domain of the literature review)	8-9
34	Guidelines for future research	8-9

From: Stroup DF, Berlin JA, Morton SC, et al, for the Meta-analysis Of Observational Studies in Epidemiology (MOOSE) Group. Meta-analysis of Observational Studies in Epidemiology. A Proposal for Reporting. *JAMA*. 2000;283(15):2008-2012. doi: 10.1001/jama.283.15.2008.

Supplementary Table2: Search strategy (MEDLINE via PubMed)

No	Search terms
#1	"Cerebrovascular Disorders"[Mesh]
#2	"Basal Ganglia Cerebrovascular Disease"[Mesh]
#3	"brain ischemia"[Mesh]
#4	"Carotid Artery Diseases"[Mesh]
#5	"Intracranial Arterial Diseases"[Mesh]
#6	"Intracranial Embolism and Thrombosis"[Mesh]
#7	"Intracranial Hemorrhages"[Mesh]
#8	"Stroke"[Mesh]
#9	#1 OR #2 OR #3 OR #4 OR #5 OR #6 OR #7 OR #8
#10	"stroke"[Title/Abstract]
#11	"cerebrovasc*"[Title/Abstract]
#12	"brain vasc*"[Title/Abstract]
#13	"cerebral vasc*"[Title/Abstract]
#14	"cva"[Title/Abstract]
#15	"apoplex*"[Title/Abstract]
#16	#10 OR #11 OR #12 OR #13 OR #14 OR #15

#17	"brain*"[Title/Abstract]
#18	"cerebr*"[Title/Abstract]
#19	"cerebell*"[Title/Abstract]
#20	"vertebrobasilar"[Title/Abstract]
#21	"hemisphere*"[Title/Abstract]
#22	"intracran*"[Title/Abstract]
#23	"intracerebral"[Title/Abstract]
#24	"infratentorial"[Title/Abstract]
#25	"supratentorial"[Title/Abstract]
#26	"MCA"[Title/Abstract]
#27	"anterior circulation"[Title/Abstract]
#28	"posterior circulation"[Title/Abstract]
#29	"basal ganglia"[Title/Abstract]
#30	#17 OR #18 OR #19 OR #20 OR #21 OR #22 OR #23 OR #24 OR #25 OR #26 OR #27 OR #28 OR #29
#31	"ischemi*"[Title/Abstract]
#32	"ischaemi*"[Title/Abstract]
#33	"infarct*"[Title/Abstract]
#34	"thrombo*"[Title/Abstract]
#35	"emboli*"[Title/Abstract]
#36	#31 OR #32 OR #33 OR #34 OR #35
#37	#30 AND #36
#38	"parenchymal"[Title/Abstract]
#39	"intraventricular"[Title/Abstract]
#40	"basal gangli*"[Title/Abstract]
#41	#17 OR #18 OR #19 OR #22 OR #23 OR #24 OR #25 OR #38 OR #39 OR #40
#42	"haemorrhage*"[Title/Abstract]
#43	"hemorrhage*"[Title/Abstract]
#44	"haematoma*"[Title/Abstract]
#45	"hematoma*"[Title/Abstract]
#46	"bleed*"[Title/Abstract]
#47	#42 OR #43 OR #44 OR #45 OR #46
#48	#41 AND #47

#49	#9 OR #16 OR #37 OR #48
#50	"Frail Elderly"[Mesh]
#51	"frail*"[Title/Abstract]
#52	"index"[Title/Abstract]
#53	"phenotype"[Title/Abstract]
#54	"assess*"[Title/Abstract]
#55	#52 OR #53 OR #54
#56	#51 AND #55
#57	"scor*"[Title/Abstract]
#58	"frail*"[Title/Abstract] AND "scor*"[Title/Abstract]
#59	"Rockwood"[Title/Abstract]
#60	"Fried"[Title/Abstract]
#61	"index*"[Title/Abstract]
#62	#51 OR #52 OR #53
#63	#60 AND #62
#64	#50 OR #51 OR #56 OR #58 OR #59 OR #63
#65	#49 AND #64
#66	Mortality[Mesh]
#67	Survival[Mesh]
#68	Death[Mesh]
#69	Length of Stay[Mesh]
#70	Patient Discharge[Mesh]
#71	Recovery of Function[Mesh]
#72	Activities of Daily Living[Mesh]
#73	#66 OR #67 OR #68 OR #69 OR #70 OR #71 OR #72
#74	mortality[Title/Abstract]
#75	surviv*[Title/Abstract]
#76	death*[Title/Abstract]
#77	los[Title/Abstract]
#78	("length of"[Title/Abstract] OR "duration of"[Title/Abstract]) AND ("stay"[Title/Abstract])
#79	("length"[Title/Abstract] OR "duration"[Title/Abstract]) AND ("stay"[Title/Abstract])
#80	bed days[Title/Abstract]

#81	("length of"[Title/Abstract] OR "duration of"[Title/Abstract] OR "days of"[Title/Abstract]) AND ("hospital*"[Title/Abstract])
#82	("length"[Title/Abstract] OR "duration"[Title/Abstract] OR "days"[Title/Abstract]) AND ("hospital*"[Title/Abstract])
#83	("inpatient"[Title/Abstract] OR "patient"[Title/Abstract] OR "short"[Title/Abstract]) AND ("stay*"[Title/Abstract] OR "throughput"[Title/Abstract] OR "flow*"[Title/Abstract] OR "days"[Title/Abstract])
#84	("discharge*"[Title/Abstract] OR "stay"[Title/Abstract]) AND ("delay*"[Title/Abstract] OR "timely"[Title/Abstract] OR "timeliness"[Title/Abstract] OR "fast"[Title/Abstract] OR "faster"[Title/Abstract] OR "sooner"[Title/Abstract] OR "quick*"[Title/Abstract] OR "haste*"[Title/Abstract] OR "rapid*"[Title/Abstract] OR "early"[Title/Abstract] OR "earlier"[Title/Abstract] OR "reduc*"[Title/Abstract] OR "decrease"[Title/Abstract] OR "lessen"[Title/Abstract] OR "speed*"[Title/Abstract])
#85	("function*"[Title/Abstract]) AND ("outcome*"[Title/Abstract] OR "recovery*"[Title/Abstract] OR "independen*"[Title/Abstract] OR "status"[Title/Abstract] OR "assess*"[Title/Abstract] OR "evaluat*"[Title/Abstract])
#86	activities of daily living[Title/Abstract]
#87	ADL[Title/Abstract]
#88	modified rankin[Title/Abstract]
#89	barthel index[Title/Abstract]
#90	#74 OR #75 OR #76 OR #77 OR #78 OR #79 OR #80 OR #81 OR #82 OR #83 OR #84 OR #85 OR #86 OR #87 OR #88 OR #89
#91	#73 AND #90
#92	"Epidemiologic Studies"[MeSH Terms] OR "epidemiologic"[Title/Abstract] OR "longitudinal"[Title/Abstract] OR "cohort"[Title/Abstract] OR "case- control"[Title/Abstract] OR "cross-sectional"[Title/Abstract] OR "follow- up"[Title/Abstract] OR "observational"[Title/Abstract] OR "prospective"[Title/Abstract] OR "retrospective"[Title/Abstract]
#93	#65 AND #91 AND #92

Supplementary Table3: Search strategy (EMBASE via Dialog)

No	Search terms
S1	EMB.EXACT.EXPLODE("cerebrovascular disease")
S2	EMB.EXACT.EXPLODE("cerebrovascular accident")
S3	S1 OR S2
S4	AB(stroke) OR TI(stroke)
S5	AB(cerebrovasc*) OR TI(cerebrovasc*)
S6	AB("brain vasc*") OR TI("brain vasc*")
S7	AB("cerebral vasc*") OR TI("cerebral vasc*")
S8	AB(cva) OR TI(cva)
S9	AB(apoplex*) OR TI(apoplex*)

S10	S4 OR S5 OR S6 OR S7 OR S8 OR S9
S11	AB(brain*) OR TI(brain*)
S12	AB(cerebr*) OR TI(cerebr*)
S13	AB(cerebell*) OR TI(cerebell*)
S14	AB(vertebrobasilar) OR TI(vertebrobasilar)
S15	AB(hemisphere*) OR TI(hemisphere*)
S16	AB(intracran*) OR TI(intracran*)
S17	AB(intracerebral) OR TI(intracerebral)
S18	AB(infratentorial) OR TI(infratentorial)
S19	AB(supratentorial) OR TI(supratentorial)
S20	AB(MCA) OR TI(MCA)
S21	AB("anterior circulation") OR TI("anterior circulation")
S22	AB("posterior circulation") OR TI("posterior circulation")
S23	AB("basal ganglia") OR TI("basal ganglia")
S24	S11 OR S12 OR S13 OR S14 OR S15 OR S16 OR S17 OR S18 OR S19 OR S20 OR S21 OR S22 OR S23
S25	AB(ischemi*) OR TI(ischemi*)
S26	AB(ischaemi*) OR TI(ischaemi*)
S27	AB(infarct*) OR TI(infarct*)
S28	AB(thrombo*) OR TI(thrombo*)
S29	AB(emboli*) OR TI(emboli*)
S30	S25 OR S26 OR S27 OR S28 OR S29
S31	S24 AND S30
S32	AB(parenchymal) OR TI(parenchymal)
S33	AB(intraventricular) OR TI(intraventricular)
S34	AB("basal gangli*") OR TI("basal gangli*")
S35	S11 OR S12 OR S13 OR S14 OR S15 OR S16 OR S32 OR S33 OR S34
S36	AB(haemorrhage*) OR TI(haemorrhage*)
S37	AB(hemorrhage*) OR TI(hemorrhage*)
S38	AB(haematoma*) OR TI(haematoma*)
S39	AB(hematoma*) OR TI(hematoma*)
S40	AB(bleed*) OR TI(bleed*)

S41	S36 OR S37 OR S38 OR S39 OR S40
S42	S35 AND S41
S43	S3 OR S10 OR S31 OR S42
S44	EMB.EXACT.EXPLODE("frail elderly")
S45	AB(frail*) OR TI(frail*)
S46	AB(index) OR TI(index)
S47	AB(phenotype) OR TI(phenotype)
S48	AB(assess*) OR TI(assess*)
S49	S45 AND (S46 OR S47 OR S48)
S50	AB(scor*) OR TI(scor*)
S51	S45 AND S50
S52	AB(Rockwood) OR TI(Rockwood)
S53	AB(Fried) OR TI(Fried)
S54	S53 AND (S46 OR S47 OR S48)
S55	S44 OR S45 OR S49 OR S51 OR S54
S56	EMB.EXACT.EXPLODE("mortality")
S57	EMB.EXACT.EXPLODE("survival")
S58	EMB.EXACT.EXPLODE("death")
S59	EMB.EXACT.EXPLODE("length of stay")
S60	EMB.EXACT.EXPLODE("hospital discharge")
S61	EMB.EXACT.EXPLODE("daily life activity")
S62	S56 OR S57 OR S58 OR S59 OR S60 OR S61
S63	(TI(mortality) OR AB(mortality)) OR (TI(death*) OR AB(death*)) OR (TI(surviv*) OR AB(surviv*)) OR (TI(los) OR AB(los)) OR (((TI("length of") OR AB("length of")) OR (TI("duration of") OR AB("duration of"))) AND (TI(stay) OR AB(stay))) OR (((TI(length) OR AB(length)) OR (TI(duration) OR AB(duration))) AND (TI(stay) OR AB(stay))) OR (TI("bed days") OR AB("bed days")) OR ((TI("length of") OR AB("length of")) OR (TI("duration of") OR AB("duration of")) OR (TI("days of") OR AB("days of"))) AND (TI(hospital*) OR AB(hospital*)) OR ((((TI(length) OR AB(length)) OR (TI(duration) OR AB(duration))) OR (TI(days) OR AB(days))) AND (TI(hospital*) OR AB(hospital*))) OR ((((TI(inpatient) OR AB(inpatient)) OR (TI(patient) OR AB(patient))) OR (TI(short) OR AB(short))) AND ((TI(stay*) OR AB(stay*)) OR (TI(throughput) OR AB(throughput)) OR (TI(flow*) OR AB(flow*)) OR (TI(days) OR AB(days)))) OR (((TI(discharge*) OR AB(discharge*)) OR (TI(stay) OR AB(stay))) AND ((TI(delay*) OR AB(delay*) OR TI(timely) OR AB(timely) OR TI(timeliness) OR AB(timeliness) OR TI(fast) OR AB(fast) OR TI(faster) OR AB(faster) OR TI(sooner) OR AB(sooner) OR TI(quick*) OR AB(quick*) OR TI(haste*) OR AB(haste*) OR TI(rapid*) OR AB(rapid*) OR TI(early) OR AB(early) OR TI(earlier) OR AB(earlier) OR

	TI(reduc*) OR AB(reduc*) OR TI(decrease) OR AB(decrease) OR TI(lessen) OR AB(lessen) OR TI(speed*) OR AB(speed*)))) OR ((TI(function*) OR AB(function*)) AND (TI(outcome*) OR AB(outcome*)) OR (TI(recovery*) OR AB(recovery*)) OR (TI(independen*) OR AB(independen*)) OR (TI(status) OR AB(status)) OR (TI(assess*) OR AB(assess*)) OR (TI(evaluat*) OR AB(evaluat*))) OR (TI("activities of daily living") OR AB("activities of daily living")) OR (TI(ADL) OR AB(ADL)) OR (TI("modified rankin") OR AB("modified rankin")) OR (TI("barthel index") OR AB("barthel index"))
S64	S62 OR S63
S65	(TI(epidemiologic) OR AB(epidemiologic) OR TI(longitudinal) OR AB(longitudinal) OR TI(cohort) OR AB(cohort) OR TI(case-control) OR AB(case-control) OR TI(cross- sectional) OR AB(cross-sectional) OR TI(follow-up) OR AB(follow-up) OR TI(observational) OR AB(observational) OR TI(prospective) OR AB(prospective) OR TI(retrospective) OR AB(retrospective))
S66	S43 AND S55 AND S64 AND S65

Supplementary Table4: Search strategy (CENTRAL)

No	Search terms
#1	MeSH descriptor: [Cerebrovascular Disorders] explode all trees
#2	MeSH descriptor: [Basal Ganglia Cerebrovascular Disease] explode all trees
#3	MeSH descriptor: [Brain Ischemia] explode all trees
#4	MeSH descriptor: [Carotid Artery Diseases] explode all trees
#5	MeSH descriptor: [Intracranial Arterial Diseases] explode all trees
#6	MeSH descriptor: [Intracranial Embolism and Thrombosis] explode all trees
#7	MeSH descriptor: [Intracranial Hemorrhages] explode all trees
#8	MeSH descriptor: [Stroke] explode all trees
#9	#1 OR #2 OR #3 OR #4 OR #5 OR #6 OR #7 OR #8
#10	(stroke):ti,ab,kw
#11	(cerebrovasc*):ti,ab,kw
#12	(brain vasc*):ti,ab,kw
#13	(cerebral vasc*):ti,ab,kw
#14	(cva):ti,ab,kw
#15	(apoplex*):ti,ab,kw
#16	#10 OR #11 OR #12 OR #13 OR #14 OR #15
#17	(brain*):ti,ab,kw
#18	(cerebr*):ti,ab,kw
#19	(cerebell*):ti,ab,kw
#20	(vertebrobasilar):ti,ab,kw

#21	(hemisphere*):ti,ab,kw
#22	(intracran*):ti,ab,kw
#23	(intracerebral):ti,ab,kw
#24	(infratentorial):ti,ab,kw
#25	(supratentorial):ti,ab,kw
#26	(MCA):ti,ab,kw
#27	(anterior circulation):ti,ab,kw
#28	(posterior circulation):ti,ab,kw
#29	(basal ganglia):ti,ab,kw
#30	#17 OR #18 OR #19 OR #20 #21 OR #22 OR #23 OR #24 OR #25 OR #26 OR #27 OR #28 OR #29
#31	(ischemi*):ti,ab,kw
#32	(ischaemi*):ti,ab,kw
#33	(infarct*):ti,ab,kw
#34	(thrombo*):ti,ab,kw
#35	(emboli*):ti,ab,kw
#36	#31 OR #32 OR #33 OR #34 OR #35
#37	#30 AND #36
#38	(parenchymal):ti,ab,kw
#39	(intraventricular):ti,ab,kw
#40	(basal gangli*):ti,ab,kw
#41	#17 OR #18 OR #19 OR #22 OR #23 OR #24 OR #25 OR #38 OR #39 OR #40
#42	(haemorrhage*):ti,ab,kw
#43	(hemorrhage*):ti,ab,kw
#44	(haematoma*):ti,ab,kw
#45	(hematoma*):ti,ab,kw
#46	(bleed*):ti,ab,kw
#47	#42 OR #43 OR #44 OR #45 OR #46
#48	#41 AND #47
#49	#9 OR #16 OR #37 OR #48
#50	MeSH descriptor: [Frail Elderly] explode all trees
#51	(frail*):ti,ab,kw

#52	(index):ti,ab,kw
#53	(phenotype):ti,ab,kw
#54	(assess*):ti,ab,kw
#55	#52 OR #53 OR #54
#56	#51 AND #55
#57	(scor*):ti,ab,kw
#58	#51 AND #57
#59	(Rockwood):ti,ab,kw
#60	(Fried):ti,ab,kw
#61	(index*):ti,ab,kw
#62	#51 OR #52 OR #53
#63	#60 AND #62
#64	#50 OR #51 OR #56 OR #58 OR #59 OR #63
#65	#49 AND #64
#66	MeSH descriptor: [Mortality] explode all trees
#67	MeSH descriptor: [Survival] explode all trees
#68	MeSH descriptor: [Death] explode all trees
#69	MeSH descriptor: [Length of Stay] explode all trees
#70	MeSH descriptor: [Patient Discharge] explode all trees
#71	MeSH descriptor: [Recovery of Function] explode all trees
#72	MeSH descriptor: [Activities of Daily Living] explode all trees
#73	#66 OR #67 OR #68 OR #69 OR #70 OR #71 OR #72
#74	(mortality):ti,ab,kw
#75	(death*):ti,ab,kw
#76	(surviv*):ti,ab,kw
#77	(los):ti,ab,kw
#78	((length of):ti,ab,kw OR (duration of):ti,ab,kw) AND ((stay):ti,ab,kw)
#79	((length):ti,ab,kw OR (duration):ti,ab,kw) AND ((stay):ti,ab,kw)
#80	(bed days):ti,ab,kw
#81	((length of):ti,ab,kw OR (duration of):ti,ab,kw OR (days of):ti,ab,kw) AND ((hospital*):ti,ab,kw)
#82	((length):ti,ab,kw OR (duration):ti,ab,kw OR (days):ti,ab,kw) AND ((hospital*):ti,ab,kw)
#83	((inpatient):ti,ab,kw OR (patient):ti,ab,kw OR (short):ti,ab,kw) AND ((stay*):ti,ab,kw OR

	(throughput):ti,ab,kw OR (flow*):ti,ab,kw OR (days):ti,ab,kw)
#84	((discharge*):ti,ab,kw OR (discharge*):ti,ab,kw) AND ((delay*):ti,ab,kw OR (timely):ti,ab,kw OR (timeliness):ti,ab,kw OR (fast):ti,ab,kw OR (faster):ti,ab,kw OR (sooner):ti,ab,kw OR (quick*):ti,ab,kw OR (haste*):ti,ab,kw OR (rapid*):ti,ab,kw OR (early):ti,ab,kw OR (earlier):ti,ab,kw OR (reduc*):ti,ab,kw OR (decrease):ti,ab,kw OR (lessen):ti,ab,kw OR (speed*):ti,ab,kw)
#85	((function*):ti,ab,kw)AND ((outcome*):ti,ab,kw OR (recovery*):ti,ab,kw OR (independen*):ti,ab,kw OR (status):ti,ab,kw OR (assess*):ti,ab,kw OR (evaluat*):ti,ab,kw)
#86	(Activities of Daily Living):ti,ab,kw
#87	(ADL):ti,ab,kw
#88	(modified rankin):ti,ab,kw
#89	(barthel index):ti,ab,kw
#90	#74 OR #75 OR #76 OR #77 OR #78 OR #79 OR #80 OR #81 OR #82 OR #83 OR #84 OR #85 OR #86 OR #87 OR #88 OR #89
#91	#73 AND #90
#92	MeSH descriptor: [Epidemiologic Studies] explode all trees OR (epidemiologic):ti,ab,kw OR (longitudinal):ti,ab,kw OR (cohort):ti,ab,kw OR (case- control):ti,ab,kw OR (cross-sectional):ti,ab,kw OR (follow-up):ti,ab,kw OR (observational):ti,ab,kw OR (prospective):ti,ab,kw OR (retrospective):ti,ab,kw
#93	#65 AND #91 AND #92

Supplementary Table5: Search strategy (CINAHL via EBSCO)

No	Search terms
S1	(MH "Cerebrovascular Disorders+")
S2	(MH "Basal Ganglia Cerebrovascular Disease+")
S3	(MH "Carotid Artery Diseases+")
S4	(MH "Intracranial Arterial Diseases+")
S5	(MH "Intracranial Embolism and Thrombosis+")
S6	(MH "Stroke+")
S7	S1 OR S2 OR S3 OR S4 OR S5 OR S6
S8	TI stroke or AB stroke
S9	TI cerebrovasc* or AB cerebrovasc*

S10	TI brain vasc* or AB brain vasc*
S11	TI cerebral vasc* or AB cerebral vasc*
S12	TI cva or AB cva
S13	TI apoplex* or AB apoplex*
S14	S8 OR S9 OR S10 OR S11 OR S12 OR S13
S15	TI brain* or AB brain*
S16	TI cerebr* or AB cerebr*
S17	TI cerebell* or AB cerebell*
S18	TI vertebrobasilar or AB vertebrobasilar
S19	TI hemisphere* or AB hemisphere*
S20	TI intracran* or AB intracran*
S21	TI intracerebral or AB intracerebral
S22	TI infratentorial or AB infratentorial
S23	TI supratentorial or AB supratentorial
S24	TI MCA or AB MCA
S25	TI anterior circulation or AB anterior circulation
S26	TI posterior circulation or AB posterior circulation
S27	TI basal ganglia or AB basal ganglia
S28	S15 OR S16 OR S17 OR S18 OR S19 OR S20 OR S21 OR S22 OR S23 OR S24 OR S25 OR S26 OR S27
S29	TI ischemi* or AB ischemi*
S30	TI ischaemi* or AB ischaemi*
S31	TI infarct* or AB infarct*
S32	TI thrombo* or AB thrombo*
S33	TI emboli* or AB emboli*
S34	S29 OR S30 OR S31 OR S32 OR S33
S35	S28 AND S34
S36	TI parenchymal or AB parenchymal
S37	TI intraventricular or AB intraventricular
S38	TI basal gangli* or AB basal gangli*
S39	S15 OR S16 OR S17 OR S20 OR S21 OR S22 OR S23 OR S36 OR S37 OR S38
S40	TI haemorrhage* or AB haemorrhage*

S41	TI hemorrhage* or AB hemorrhage*
S42	TI haematoma* or AB haematoma*
S43	TI hematoma* or AB hematoma*
S44	TI bleed* or AB bleed*
S45	S40 OR S41 OR S42 OR S43 OR S44
S46	S39 AND S45
S47	S7 OR S14 OR S35 OR S46
S48	(MH "Frail Elderly")
S49	TI frail* or AB frail*
S50	TI index or AB index
S51	TI phenotype or AB phenotype
S52	TI assess* or AB assess*
S53	S50 OR S51 OR S52
S54	S49 AND S53
S55	TI scor* or AB scor*
S56	S49 AND S55
S57	TI Rockwood OR AB Rockwood
S58	TI fried OR AB fried
S59	TI index* OR AB index*
S60	S49 OR 51 OR S59
S61	S58 AND S60
S62	S48 OR S49 OR S54 OR S56 OR S57 OR S61
S63	S47 AND S62
S64	(MH "Mortality+")
S65	(MH "Survival")
S66	(MH "Death+")
S67	(MH "Length of Stay")
S68	(MH "Patient Discharge+")
S69	(MH "Activities of Daily Living+")
S70	S64 OR S65 OR S66 OR S67 OR S68 OR S69
S71	TI Mortality OR AB Mortality
S72	TI death* OR AB death*

S73	TI surviv* OR AB surviv*
S74	TI los OR AB los
S75	((TI length of OR AB length of)) OR (TI duration of OR AB duration of)) AND((TI stay OR AB stay))
S76	((TI length OR AB length) OR (TI duration OR AB duration)) AND ((TI stay OR AB stay))
S77	TI bed days OR AB bed days
S78	((TI length of OR AB length of) OR (TI duration of OR AB duration of) OR (TI days of OR AB days of)) AND ((TI hospital* OR AB hospital*))
S79	((TI length OR AB length) OR (TI duration OR AB duration) OR (TI days OR AB days")) AND ((TI hospital* OR AB hospital*))
S80	((TI inpatient OR AB inpatient) OR (TI patient OR AB patient) OR (TI short OR AB short)) AND ((TI stay* OR AB stay*) OR (TI throughput OR AB throughput) OR (TI flow* OR AB flow*) OR (TI days OR AB days))
S81	((TI discharge* OR AB discharge*) OR (TI stay OR AB stay)) AND ((TI delay* OR AB delay*) OR (TI timely OR AB timely) OR (TI timeliness OR AB timeliness) OR (TI fast OR AB fast) OR (TI faster OR AB faster) OR (TI sooner OR AB sooner) OR (TI quick* OR AB quick*) OR (TI haste* OR AB haste*) OR (TI rapid* OR AB rapid*) OR (TI early OR AB early) OR (TI earlier OR AB earlier) OR (TI reduc* OR AB reduc*) OR (TI decrease OR AB decrease) OR (TI lessen OR AB lessen) OR (TI speed* OR AB speed*))
S82	((TI function* OR AB function*)) AND ((TI outcome* OR AB outcome*) OR (TI recovery* OR AB recovery*) OR (TI independen* OR AB independen*) OR (TI status OR AB status) OR (TI assess* OR AB assess*) OR (TI evaluat* OR AB evaluat*))
S83	TI Activities of Daily Living OR AB Activities of Daily Living
S84	TI ADL OR AB ADL
S85	TI modified rankin OR AB modified rankin
S86	TI barthel index OR AB barthel index
S87	S71 OR #72 OR S73 OR S74 OR S75 OR S76 OR S77 OR S78 OR S79 OR S80 OR S81 OR S82 OR S83 OR S84 OR S85 OR S86
S88	S70 AND S87
S89	(TI epidemiologic OR AB epidemiologic) OR (TI longitudinal OR AB longitudinal) OR (TI cohort OR AB cohort) OR (TI case-control OR AB case- contro) OR (TI cross-sectional OR AB cross-sectional) OR (TI follow-up OR AB follow-up) OR (TI observational OR AB observational) OR (TI prospective OR AB prospective) OR (TI retrospective OR AB retrospective)
S90	S63 AND S88 AND S89

Supplementary Table6: Search strategy (ICTRP)

No	Search terms
#1	"stroke" OR "cerebrovasc*" OR "brain vasc*" OR "cerebral vasc*" OR "CVA" OR "appoplex*” OR "ischemi*" OR "ischaemi*" OR "infarct*" OR "thrombo*" OR "emboli*” OR "haemorrhage*" OR "hemorrhage*" OR "haematoma*" OR "hematoma*" OR "bleed*"
#2	“frail” OR “frailty”
#3	#1 AND #2 Recruitme¥t status: All

Supplementary Table7: Search strategy (Clinicaltrials.gov)

No	Search terms
#1	"stroke" OR "cerebrovascular" OR "brain vascular" OR "cerebral vascular" OR "CVA" OR "appoplex” OR "ischemic" OR "ischemia" OR "ischaemic" OR "ischaemia" OR "infarction" OR "thrombosis" OR "emboliosm” OR "haemorrhage" OR "hemorrhage" OR "haematoma" OR "hematoma" OR "bleed"
#2	“frail” OR “frailty”
#3	#1 AND #2 Study status: All studies

Supplementary Table8: Excluded studies and reasons for exclusion

Reason for exclusion: Wrong exposure
Dalton et al. (2023) International Journal of Stroke 18 (2), 52-53.
Reason for exclusion: Wrong outcome
Fernandes et al. (2023) International Journal of Stroke 18 (3).<br>Reehal et al. (2023) International Journal of Stroke 18 (3), 387-388.<br>Colantonio et al. (1996) Archives of physical medicine and rehabilitation 77 (6), 562-6.<br>Winovich et al. (2017) Stroke 48 (7), 1818-1826.<br>Kanai et al. (2020) J Stroke Cerebrovascular Diseases 29 (12).
Reason for exclusion: Wrong design
Alicia et al. (2022) Revista Cientifica de la Sociedad Espanola de Enfermeria Neurologica 56,29-36.<br>Baztán et al. (2009) Gerontology 55 (3), 269-74.<br>Buchman et al. (2008) Neurology 71 (7), 499-504.<br>Fearon et al. (2013) Cerebrovascular Diseases 35, 311.<br>Fearon et al. (2012) Stroke 43 (12), 3184-8.<br>Hanlon et al. (2023) International Journal of Stroke 18 (6), 720-727.<br>Longstreth et al. (2001) Neurology 56 (3),368-75.<br>Miranda et al. (2024) Frontiers in Aging Neuroscience 16.<br>Myint et al. (2017) Age and ageing 46 (1), 83-90.
Reason for exclusion: Wrong population
Annie et al. (2017) Journal of the American College of Surgeons 225 (5), 658-665.<br>Ekerstad et al. (2011) Circulation 124 (22), 2397-404.<br>Hartley et al. (2023) International Journal of Stroke 18 (1), 62-63.<br>Lightbody et al. (2002) Evidence-based Mental Health 5 (4), 109.<br>Nozo et al. (2022) Geriatrics & Gerontology International 22 (8), 642-647.<br>Stortecky et al. (2012) JACC. Cardiovascular interventions 5 (5), 489-496.

Reason for exclusion: Wrong control
Matthew et al. (2023) European Stroke Journal 8 (2), 120.
Reason for other
Mohammed et al. (2022) Cerebrovascular Diseases 51, 88.<br>Sivakumar et al. (2018) European Stroke Journal 3 (1), 368-369.

Supplementary Table9: Confounders of the included studies

Auter/Year	Confounders
Mennema,2023[21)]	Nr
Joyce,2022[22)]	age, gender, thrombolytic therapy, NIHSS at admission
Yang,2022[23)]	age, gender, Stroke risk factors
Pilotto,2022[24)]	gender, NIHSS, vascular risk factors, treatment employed, door-to-needle time
Schnieder,2021[25)]	Nr
Evans,2020[26)]	vascular risk factors, NIHSS at admission, door-to-needle time
O'Caoimh,2024[27)]	age, gender, NIHSS at admission
Tiainen,2022[28)]	age, gender, early signs of ischemia over 1/3
Iwasawa,2023[7)]	Nr
Tan,2022[29)]	age, NIHSS, door-to-needle time
Seamon,2019[30)]	Nr
Noguchi,2021[31)]	Nr
Miranda,2022[32)]	Nr
Schnieder,2022[33)]	Nr

NIHSS：national institutes of health stroke scale　Nr：not reported

Supplementary Table10: Risk of bias assessment in studies included in the meta-analyses

Author (Year)	Study participation	Study attrition	Prognostic factor measurement	Outcome measurement	Study confounding	Statistical analysis and reporting	Overall
Mennema (2023)[21)]	Low	Low	Low	Low	Moderate	Low	Low
Joyce (2022)[22)]	Low	High	Low	Low	Low	Low	High
Yang (2022)[23)]	Low	Low	Low	Low	Low	Low	Low
Pilotto (2022)[24)]	Low	High	Low	Low	Low	Low	High
Schnieder (2021)[25)]	Low	High	Low	Low	Moderate	Low	High
Evans (2020)[26)]	Low	Moderate	Low	Low	Low	Moderate	Moderate
O'Caoimh (2024)[27)]	Low	Moderate	Low	Low	Low	Low	Low
Tiainen (2022)[28)]	Low	Moderate	Low	Low	Moderate	Low	Moderate
Iwasawa (2023)[7)]	Low	Low	Low	Low	Moderate	Low	Low
Tan (2022)[29)]	Low	Moderate	Low	Low	Low	Low	Low
Seamon (2019)[30)]	Low	Moderate	Low	Low	Low	Low	Low
Noguchi (2021)[31)]	Low	Low	Low	Low	Low	Low	Low
Miranda (2022)[32)]	Low	Low	Moderate	Low	Moderate	Moderate	High
Schnieder (2022)[33)]	Moderate	High	Moderate	Low	High	Moderate	High

Risk of bias was assessed using the Quality In Prognosis Studies (QUIPS) tool.

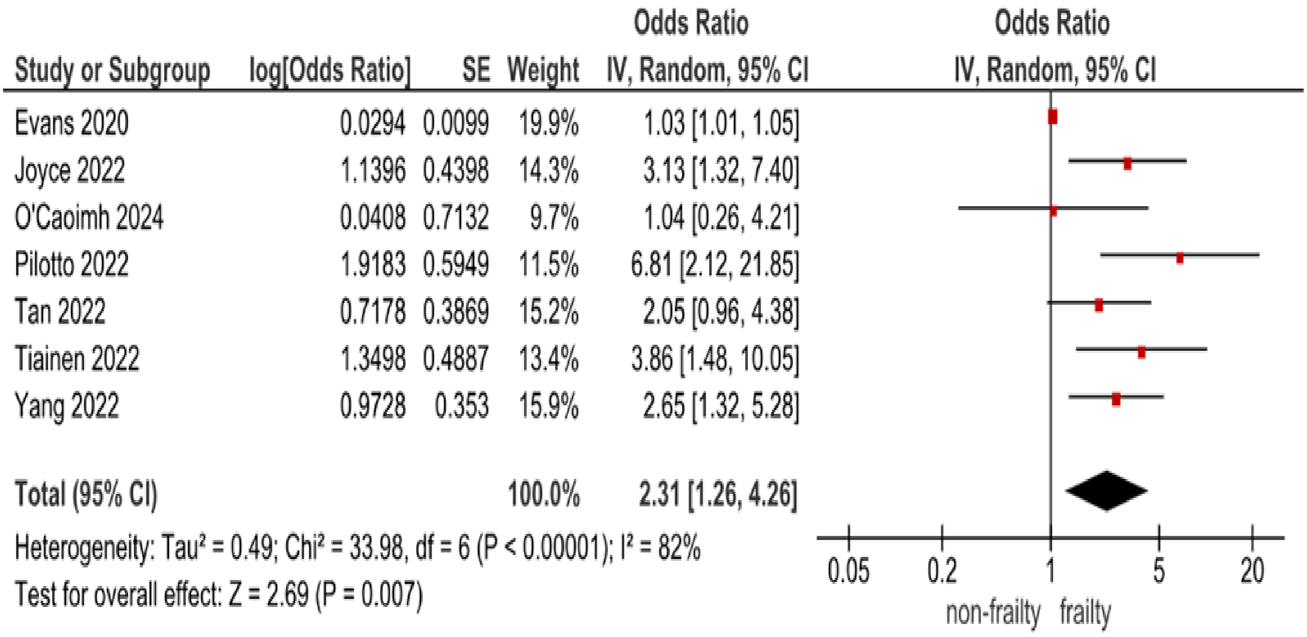

Supplementary Figure1: Sensitivity analysis of the association between pre-stroke frailty and mortality excluding studies that reported crude ORs

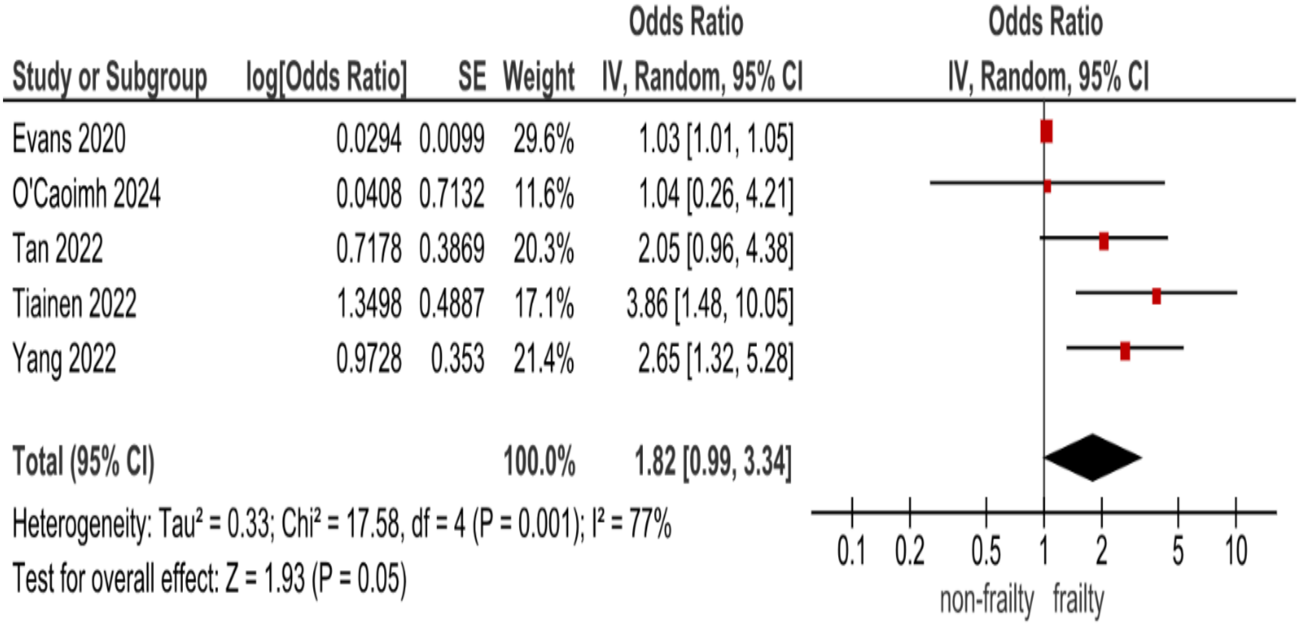

Supplementary Figure2: Sensitivity analysis of the association between pre-stroke frailty and mortality excluding studies reporting a high overall risk of bias

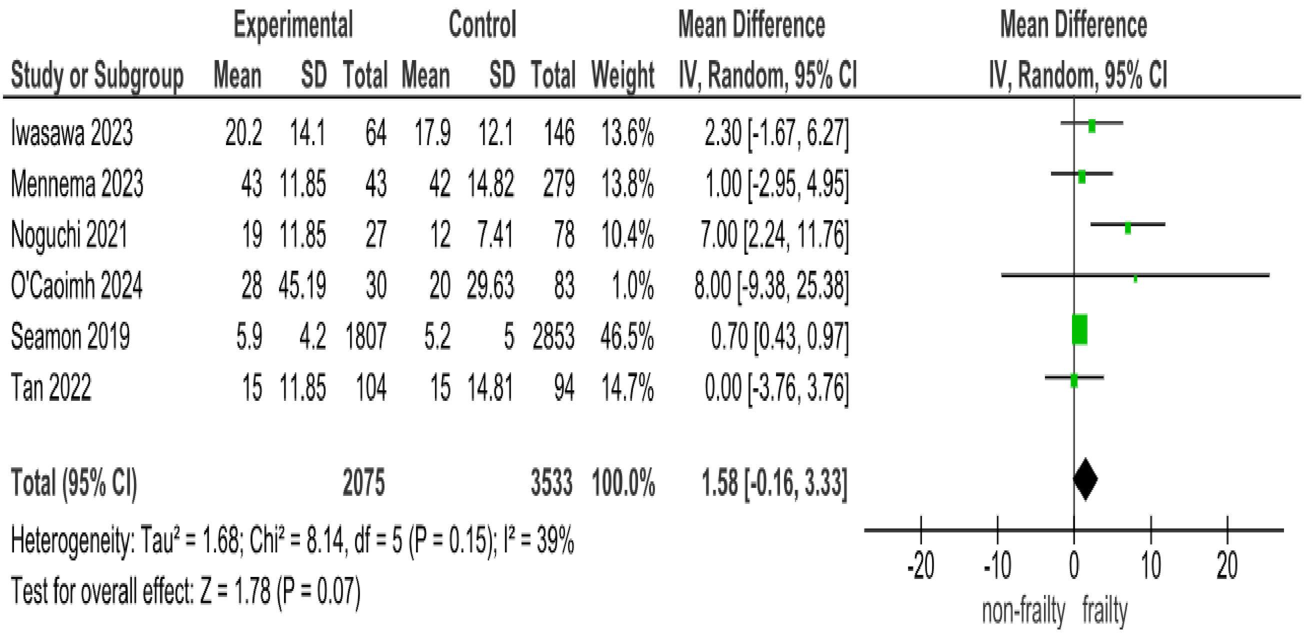

Supplementary Figure3: Sensitivity analysis of the association between pre-stroke frailty and LOS excluding studies reporting a high overall risk of bias

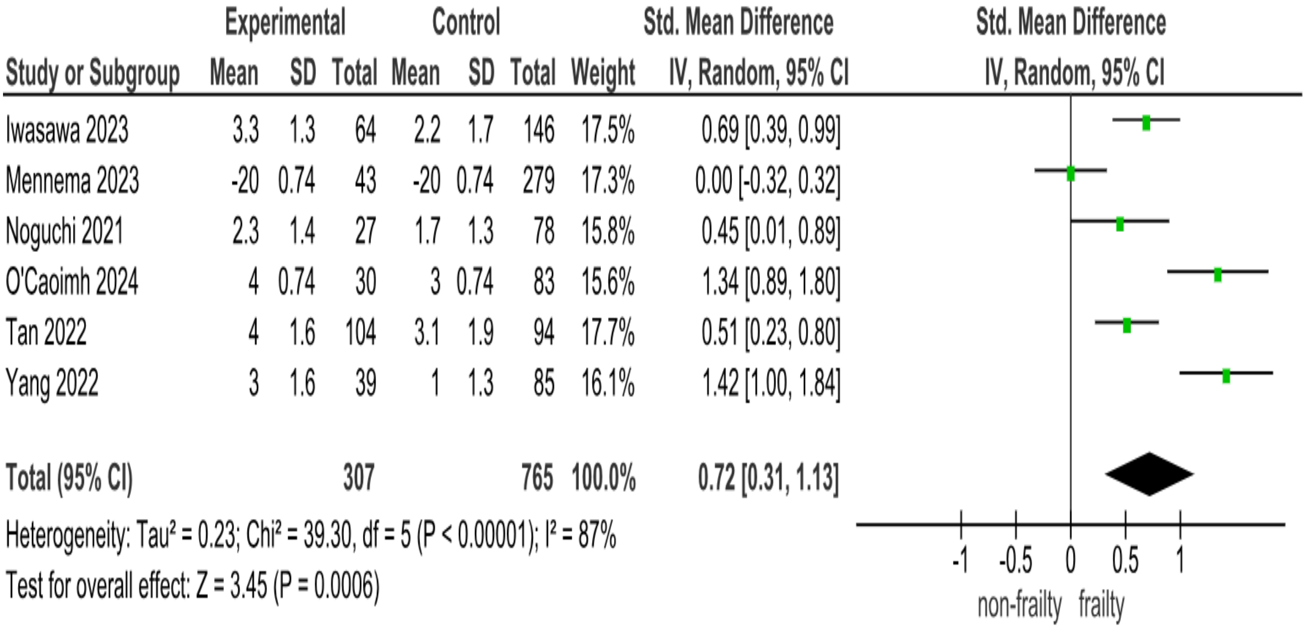

Supplementary Figure4: Sensitivity analysis of the association between pre-stroke frailty and functional outcome excluding studies reporting a high overall risk of bias
